# Histological and transcriptome analyses of testes from Duroc and Meishan boars

**DOI:** 10.1038/srep20758

**Published:** 2016-02-11

**Authors:** Haisheng Ding, Yan Luo, Min Liu, Jingshu Huang, Dequan Xu

**Affiliations:** 1 Key Laboratory of Swine Genetics and Breeding of Ministry of Agriculture, and Key Lab of Agricultural Animal Genetics, Breeding and Reproduction of Ministry of Education, Huazhong Agricultural University, Wuhan 430070, China; 2 Animal Husbandry and Veterinary Bureau of Hubei Province, Wuhan 430064, China

## Abstract

Meishan boars are known for their early sexual maturity. However, they exhibit a significantly smaller testicular size and a reduced proportion of Sertoli cells and daily sperm production compared with Duroc boars. The testes of Duroc and Meishan boars at 20, 75 and 270 days of age were used for histological and transcriptome analyses. Haematoxylin-eosin staining was conducted to observe histological structure of the testes in Duroc and Meishan boars at different ages. Although spermatogenesis occurred prior to 75 days in Meishan boars, the number of spermatogonia and Sertoli cells in Meishan boars were less than in Duroc boars at adulthood. The diameters of the seminiferous tubules of the testes differed significantly during the initiation of development of the seminiferous tubules between the two breeds. We obtained differentially expressed functional genes and analysed seven pathways involved in male sexual maturity and spermatogenesis using RNA-seq. We also detected four main alternative splicing events and many single nucleotide polymorphisms from testes. Eight functionally important genes were validated by qPCR, and Neurotrophin 3 was subjected to quantification and cellular localization analysis. Our study provides the first transcriptome evidence for the differences in sexual function development between Meishan and Duroc boars.

Different breeds of boars have different sexual function and sperm production capacities. The testis size, proportion of Sertoli cells and daily sperm production of Chinese Meishan boars is significantly smaller and lower than those of Duroc and Large White boars^1^. The onset of puberty in Chinese Meishan boars occurs at a significantly younger age (56–84 days) than conventional boars (120–180 days); Meishan boars also have lower testes weight (30–60 g paired testes weight) than conventional boars (160–240 g paired testes weight) at puberty^1^. The testes of mature Meishan boars are approximately half the size of those of Duroc and Large White boars due to the presence of fewer Sertoli cells, and Meishan boars accumulate Sertoli cells and seminiferous tubules at a more rapid rate compared with White Composite boars^1^,^2^,^3^. Earlier boar puberty can provide economic benefits because boar exposure can accelerate the utilization of replacement gilts by stimulating sexual maturation. The age at puberty determines the lifetime performance of female pigs because it affects the age at first mating, the farrowing rate, and the lifetime piglets that are born alive^4^,^5^. The high endocrine secretion and physiological attributes of Chinese Meishan boars makes these boars highly prolific, which renders them a valuable animal model for examining the mechanism of sexual development and sperm production of boars.

As an important male reproductive and endocrine organ, the testis is a critical tissue for spermatogenesis. The structure and function of the testis undergoes many changes during the initiation of the first wave of spermatogenesis and the process of sexual maturity. The organogenesis of a functional testis lays the foundation for male fertility and perpetuation of each species^6^. The functional testis primarily consists of seminiferous tubules and leydig tissues. The seminiferous tubule is composed of germ cells, including spermatogonia, primary spermatocytes, secondary spermatocytes and spermatids and Sertoli cells. The diameter and number of seminiferous tubules, the number of interstitial and germ cells, the appearance of elongate spermatids, and spermatozoa in the seminiferous tubules can affect the initiation of male puberty development and normal spermatogenesis and sperm function, which alters male fertility^7^,^8^,^9^. Sertoli cells are somatic cells that support and nurture germ cells during spermatogenesis and eventually initiate the event of germ cell differentiation, meiosis and transformation into spermatozoa. The number of Sertoli cells determines the number of germ cells that can be supported throughout spermatogenesis and the degree of sperm production^10^. Spermatogenesis is a complicated process that includes mitosis, meiosis and differentiation, in which post-meiotic male germ cells differentiate into mature spermatozoa. Spermatogenesis is a precisely regulated process in which germ cell closely interact with Sertoli cells^11^. Eventually germ cells, Sertoli cells and Leydig cells simultaneously accomplish the function of the testis.

Transcriptome sequencing (RNA-seq) is a novel, high-throughput, and deep-sequencing technology that is extensively applied in genomics research; it provides new strategies for the analysis of the functional complexity of transcriptomes^12^. RNA-seq also provides strong coverage and sensitivity and enables comprehensive analysis of gene isoforms, novel transcripts, spliceosomes and single nucleotide polymorphisms (SNPs). Transcriptomic analyses of pig reproductive organs have demonstrated the effectiveness of RNA-seq in these tissues^13^,^14^,^15^.

Meishan pigs are one of the most prolific breeds of pig in the world. Duroc sires are utilized most frequently as a Terminal/Paternal sire in a terminal cross-breeding program. In order to reveal the histologic features and molecular mechanism that underlies the earlier sexual maturity of Meishan boars and the differences in sperm production among breeds, we performed a histological comparison by haematoxylin-eosin (HE) staining of paraffin sections and analysed the number of spermatogonia and Sertoli cells and diameters of the seminiferous tubules between the testes of Meishan and Duroc boars. We also conducted transcriptome analyses of the testes of Duroc and Chinese Meishan boars using RNA-seq technology, by which we identified differential expression of genes and gene response pathways, performed quantification and cellular localization analyses, and detected alternative splicing events and single nucleotide polymorphisms. Our results have shed new light on the boar sexual maturity and sperm production and provide new approaches for the development and utilization of superior genetic resources, and improve boar fertility which is important for pig industry.

## Results

### Histological analysis of the testes of Duroc and Chinese Meishan boars

**Figure 1:**
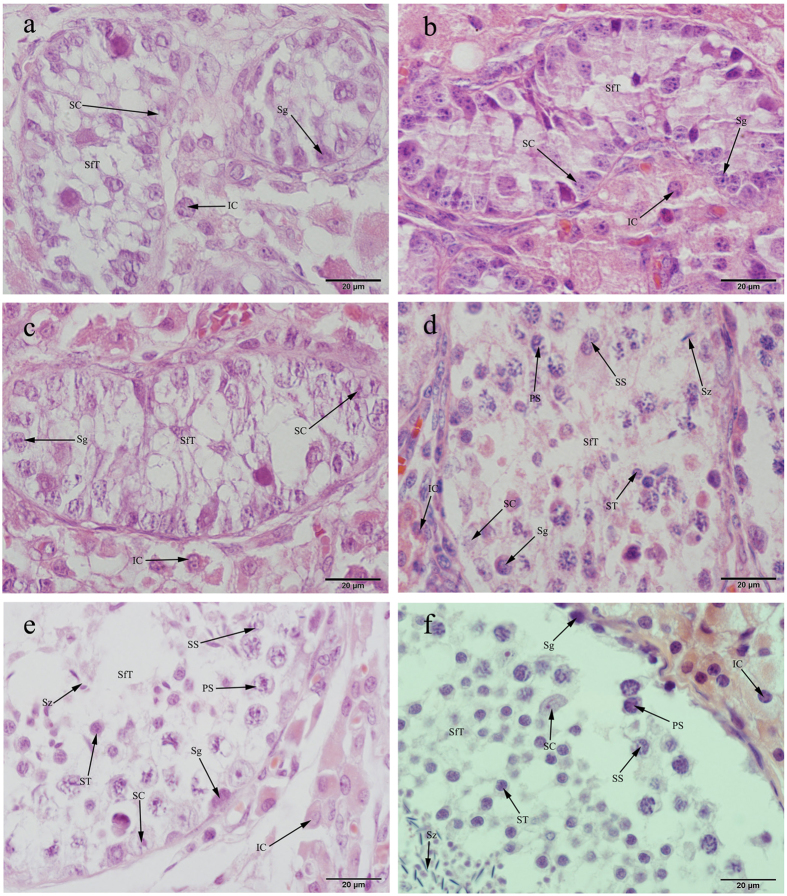
The histological observation of testes in the Duroc and Meishan boars at different ages. (**a**) Testis of 20-day-old Duroc boar. (**b**) Testis of 20-day-old Meishan boar. (**c**) Testis of 75-day-old Duroc boar. (**d**) Testis of 75-day-old Meishan boar. (**e**) Testis of 270-day-old Duroc boar. (**f**) Testis of 270-day-old Meishan boar. Bars = 20 μm. The blue parts represent the nucleus, stained by Hematoxylin Staining Solution, and the red parts represent the cytoplasm, stained by Eosin Staining Solution. SfT, seminiferous tubule; SC, Sertoli cell; IC, interstitial cell; Sg, spermatogonia; PS, primary spermatocyte; SS, secondary spermatocyte; ST, spermatid; Sz, sperm.

To gain a better understanding of testes development, HE staining analysis was conducted to compare testes and seminiferous tubules development. The testes tissues of Duroc and Meishan boars were collected at 20, 75 and 270 days. The morphological observation of the testes is showed in Fig. 1. Although few spermatogonia and Sertoli cells were found in 20-day-old Duroc and Meishan boars testes tissues, a larger number of spermatogonia and Sertoli cells were observed in the Meishan testes tissues. A few primary spermatocytes, secondary spermatocytes and spermatids with nuclear fission were detected in the seminiferous tubules of 75-day-old Meishan boars but were not observed in 75-day-old Duroc boars. A larger number of spermatogonia appeared in the seminiferous tubules in the 270-day-old Duroc and Meishan boars. We also detected a large number of primary spermatocytes, secondary spermatocytes and spermatids. Thus, sperm production occurred earlier in the Meishan boars, and puberty began at a much younger age in the Meishan boars compared with the Duroc boars.

**Figure 2:**
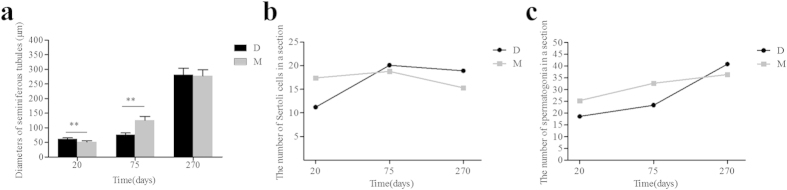
Analyses of the diameters of seminiferous tubules and the number of cells. (**a**) The diameter determination of seminiferous tubules in Duroc and Meishan boars testes at different ages (T-test, **p < 0.01). D represents Duroc boars and M represents Meishan boars. (**b**) The number of the Sertoli cells in Duroc and Meishan boars testes at different ages. (**c**) The number of spermatogonia in Duroc and Meishan boars testes at different ages.

A detailed analysis was performed to measure the diameter of 30 seminiferous tubules from the two breeds. Three boars from each breed were used; the measurements are shown in Fig. 2a. The diameters of the seminiferous tubules from the Meishan boars were significantly smaller than those from the Duroc boars on day 20 (P < 0.01). However, the diameters of the seminiferous tubules of the Meishan boars were significantly larger than those of the Duroc boars on day 75 (P < 0.01), and the diameters of the seminiferous tubules of the Meishan boars was similar to those of the Duroc boars on day 270 (P > 0.05). The diameters of the seminiferous tubules from the two breeds became significantly enlarged with an increase in growth (P < 0.01). The results demonstrated that the diameters of the seminiferous tubules from Meishan and Duroc boars increased as the animals aged from youths to adults; however, the seminiferous tubule development occurred earlier in the Meishan boars.

The number of spermatogonia and Sertoli cells was also measured from 30 seminiferous tubule cross-sections, which were collected from testes tissues from three Duroc and Meishan boars. Figure 2b indicates that a larger number of Sertoli cells were observed in the 20-day-old Meishan boars compared with Duroc boars (p < 0.01). The cell number for the Meishan boars remained stable from 20 to 75 days and decreased from 75 to 270 days. A slight difference was observed between the two breeds at 75 days (p > 0.05). However, the number of Sertoli cells in 270-day-old Duroc boars was significantly higher than in the Meishan boars (p < 0.01). The number of spermatogonia was significantly more in the Meishan boars at 20 and 75 days old compared with the Duroc boars (p < 0.01) at the same stage. But the spermatogonia was significantly more in the Duroc boars at 270 days compared with the Meishan boars (p < 0.01), as shown in Fig. 2c. We found that the spermatogonia continuously proliferated from 20 to 270 days. The number of the Sertoli cells remained stable when they attained a certain level but subsequently declined.

### Analysis of RNA-Seq Data

In this study, six cDNA libraries (M20, M75, M270, D20, D75 and D270) were constructed using total RNA from 20-, 75-, 270-day-old Meishan and Duroc boars. To ensure that the RNA-seq data satisfied the criteria for genome-wide transcriptomic analysis, we conducted standard analyses for quality control^16^ (see Supplementary Table S2 online). A total of 240,214,060 and 230,924,030 clean reads were obtained by RNA-seq from the testes of the Duroc and Meishan boars, respectively (see Supplementary Table S2b online). A total of 137,102,991 reads and 117,500,211 reads mapped to the porcine reference genome (see Supplementary Table S2c online). More than 75 M of sequence data were obtained from each sample. Raw reads that contain adaptor sequences and N >10% were removed, and the sequence data were filtered for low-quality reads at high level of stringency (see Supplementary Fig. S1 online). These steps produced more than 70 M high-quality reads, which include 88.19–95.49% of the raw reads (see Supplementary Table S2b online). Then, the clean reads were mapped to the reference genome and reference gene; approximately 55% of the reads were aligned to the reference genome and the reference gene of pig (see Supplementary Table S2c and S3 online). We measured the gene expression levels in reads per kilobase of gene per million reads (RPKM)^17^. Based on this analysis, the gene expression levels were classified into eight categories; they are provided in Supplementary Fig. S2 online. The largest proportion of genes exhibited low expression (0 < RPKM < 5), followed by moderate expression (10 < RPKM < 50); a small fraction of the genes was expressed at extremely high levels (RPKM > 500). This analysis demonstrated that high-throughput sequencing had the advantage of detecting low-abundance genes. This study detected a large fraction of genes with low expression (0 < RPKM < 10) between the Duroc and Meishan boars, which confirmed the advantage of this approach.

### Differentially expressed genes (DEGs) between Meishan and Duroc boars

**Figure 3:**
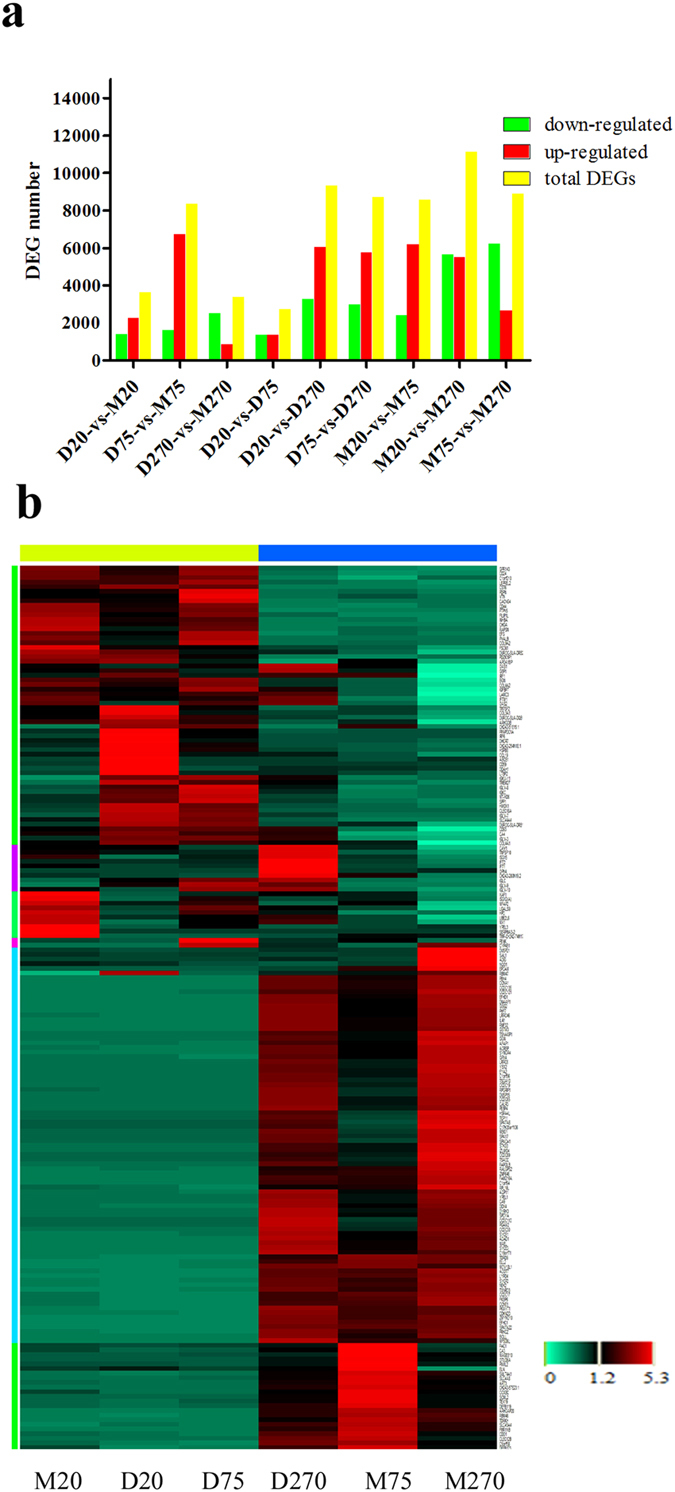
Analysis of differentially expressed genes. (**a**) DEGs (FDR ≤ 0.001 and |log2 Ratio| ≥1) detected in each sample. (**b**) The heatmap of the subset DEGs (FDR ≤ 0.001, |log2Ratio|≥3, RPKM > 1) in different samples. Six color parts in the left represent six expression patterns.

After the standard analysis, 20,524 genes were detected from the six samples and 2,724–11,111 significant differentially expressed genes (DEGs) (FDR ≤ 0.001, |log2Ratio|≥1) were filtered from each comparison, as presented in Fig. 3a. For example, the number of significant DEGs between the two breeds from the 75-day time point was substantially higher than in the 20- and 270-day time points. These results indicated significant differences in testicular development between the Duroc and Meishan boars at the age of 75 days. A greater number of significant DEGs were detected within-breed than between the breeds, and between 20 days and 75 days in Meishan boars than that in Duroc boars. This finding suggested that Meishan testicular development occurred at a faster rate than Duroc testicular development from 20 to 75 days.

Next, we filtered significant DEGs (FDR ≤ 0.001, |log2Ratio|≥3, RPKM > 1) between the breeds at the three developmental stages (20 days, 75 days and 270 days); the significant DEGs were identified using an R package. Figure 3b shows 190 genes that were filtered from the three testes developmental stages of Meishan and Duroc boars. The heat map shows two main sample branches (M20, D20 and D75 *versus* D270, M75 and M270) and six significant DEGs branches (the different color parts represent different branches). It showed that the expression pattern of D75 was similar to that of D20 and M20, while the expression pattern of D270 was similar to that of M75 and M270, which was consistent with the characteristics of testicular development between the two breeds. The genes that are represented by the fifth and sixth patterns were down-regulated in D20, D75 and M20 but up-regulated in D270, M75 and M270. The genes indicated in the sixth pattern were significantly higher in M75. These patterns may represent the genes that induced difference between M75 and D75 explain why testicular development occurred earlier in Meishan boars and why Meishan boars entered puberty before they were 75 days old. However, the genes in the first and second patterns were down-regulated in D270, M75 and M270. D270 had more up-regulated genes compared with M270; these genes may be related to the sperm production capacity as Duroc boars have a higher sperm production capacity when they reach adulthood. These results also revealed that large differences existed in the process of testicular development between the Meishan and Duroc boars.

### KEGG Analysis for DEGs and Epigenetic Regulation for Spermatogenesis

In this study, seven important pathways related to the regulation of male sexual function were identified (Table 1). The seven pathways included the largest number of DEGs that were annotated between M75 and D75, which demonstrated the large difference in testicular development between these groups. The gonadotropin-releasing hormone (GnRH) signaling pathway serves a crucial role in the regulation of mammalian reproduction. GnRH is a gonadotropin that is considered to be the main regulator of the reproductive system. GnRH can trigger the release and synthesis of luteinizing hormone (LH) and follicle-stimulating hormone (FSH). These hormones regulate the majority of the reproductive functions in both sexes^18^. We annotated the GnRH signaling pathway. As shown in Fig. S3a, some of the 29 genes at the centre of GnRH signaling were included in the list of significant DEGs. The majority of the GnRH-regulated genes were significantly down-regulated in the samples. Some family-wide DEGs in our RNA-Seq data were involved in epigenetic regulation, which was defined as non-genetic alterations to biological adaptability to environmental stimuli^19^. In mammals, five DNMTs (the DNA (cytosine-5) –methyltransferases DNMT1, DNMT2, DNMT3A, DNMT3B and DNMT3L) have been shown to regulate the demethylation or *de novo* methylation of regulatory sequences to activate and repress genes during cellular differentiation^20^. In this study, we demonstrated that multiple genes encoding DNA methyltransferases, which are responsible for catalyzing epigenetic regulation, were significantly differentially expressed in the samples and may be involved in the regulation of spermatogenesis. The expression of multiple genes, which is important for epigenetic regulation, was significantly increased in M20, D75 and D20 and significantly decreased in M270, M75 and D270; these genes included the DNA methylatransferase genes DNMT3A and DNMT3L and O-6-methylguanine-DNA methyltransferase (MGMT). Our data also showed that DNMT3B was expressed at a higher level in M270, M75 and D270 (see Supplementary Fig. S3b online). A previous study showed that the DNMT3A and DNMT3B isoforms were detected in round spermatids but were almost undetectable in residual bodies/elongating spermatids; in addition, the highest expression was observed in type A spermatogonia during the meiosis prophase^20^. These data revealed the tightly regulated expression of these genes during spermatogenesis and suggested that DNMTs may regulate sperm production.

### Alternative Splicing and single nucleotide polymorphisms (SNPs) analyses

Alternative splicing and SNPs can increase protein complexity. Although alternative splicing is widespread in eukaryotes, we may underestimate its proportion. In this study, splice sites of transcripts and various splicing events were detected using TopHat software^21^ (see Supplementary Fig. S4 online). Four primary alternative splicing events were detected: exon skipping(ES), intron retention(IR), alternative 5′ splice site(A5SS), alternative 3′ splice site(A3SS). A total of 14,819, 13,164, 20,583, 13,465, 35,468 and 17,056 alternative splicing events were detected in D20, D75, D270, M20, M75 and M270, respectively. A3SS was most ubiquitous in D20, D75, D270, M75 and M270, whereas IR frequently occured in M20. To identify SNPs in genes, we compared the sequence data with known reference sequences using SOAPsnp software and then compared the consensus sequence with the reference sequence. A total of 188,708 and 324,334 SNPs were detected from the testes of Duroc and Meishan boars, respectively. A larger number of SNPs were identified from Meishan boars than Duroc boars at the same stage (see Supplementary Fig. S5 online), which delineated the differences in the sexual function of the two breeds.

### New differentially expressed transcripts and DEGs in QTL regions related to reproduction

We identified many new differentially expressed transcripts and genes in quantitative trait locus (QTL) region that are related to reproduction. Table 2a shows 6807 new differentially expressed transcripts and 979 differentially expressed genes in the age at puberty (AGEP) region, which is related to puberty, or at its boundary. A total of 1057 new differentially expressed transcripts and 198 DEGs were detected in epididymis weight (EPIDW) region, which was related to epididymis weight. Next, we obtained 223–843 significant DEGs and 711–5,339 new differentially expressed transcripts, which were located in the reproductive QTL regions from each comparison; they are presented in Table 2b. These new transcripts and DEGs are potentially involved in boar reproduction and warrant future investigation.

### Verification of DEGs with Quantitative Real-Time PCR (qRT-PCR)

**Figure 4:**
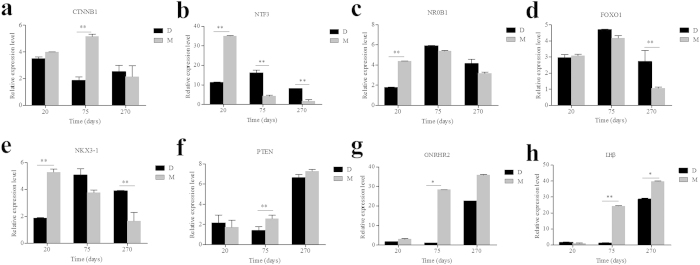
Validation of the DEGs by qRT-PCR. Comparisons of expression levels of the DEGs between Meishan (gray bars) and Duroc (black bars) boars at three periods. β-actin was used as a reference gene for normalization of qRT-PCR data. Bars represent the standard error (n = 3). The x-axis indicates age of samples. The y-axis shows the relative expression level of genes. (**a**) CTNNB1. (**b**) NTF3. (**c**) NR0B1. (**d**) FOXO1. (**e**) NKX3-1. (**f**) PTEN. (**g**) GNRHR2. (**h**) LHβ. **p < 0.01, *p < 0.05.

To evaluate our DEGs library, the expression levels of eight DEGs, which were primarily involved in boar sexual function and reproduction, were analysed by quantitative real-time PCR (qRT-PCR). Figure 4 shows the same expression tendency that was obtained with the RNA-seq analysis. The catenin (cadherin- associated protein), beta1(CTNNB1) gene was differentially expressed between M75 and D75 and was also differentially expressed during the three stages in the Meishan boars. Neurotrophin 3 (NTF3) was differentially expressed between the breeds at all three stages. The two genes serve important roles in gonad development. The nuclear receptor subfamily 0, group B, member 1(NR0B1) gene was expressed at a high level in the Meishan and Duroc boars at 75 days. The forkhead box O1(FOXO1) gene was significantly differentially expressed in D270 and M270 and was also significantly differentially expressed at the three stages in the Meishan boars. FOXO1 and NR0B1 are necessary for the process of spermatogenesis. NK3 homeobox 1 (NKX3-1) expression decreased from youths to adults in the Meishan boars and was abundantly expressed in D75. Conversely, the expression level of the phosphatase and tensin homolog (PTEN) increased with age and was highly expressed in the two breeds at 270 days. The expression of the GnRH (type 2) receptor 2 (GnRHR2) and LH beta subunit (LHβ) genes increased from 20 to 270 days between the Meishan and Duroc boars. GnRHR2 was expressed at a higher level at different stages in the Meishan compared with the Duroc boars, especially at 75 days. LHβ was highly expressed in M75 and M270, whereas a decrease in the level of expression was revealed in D75 and a high level was detected in D270. The results were consistent with the RNA-seq data in Fig. S6 and demonstrated the reliability of our data.

### Quantification and cellular localization of the boar NTF3

**Figure 5:**
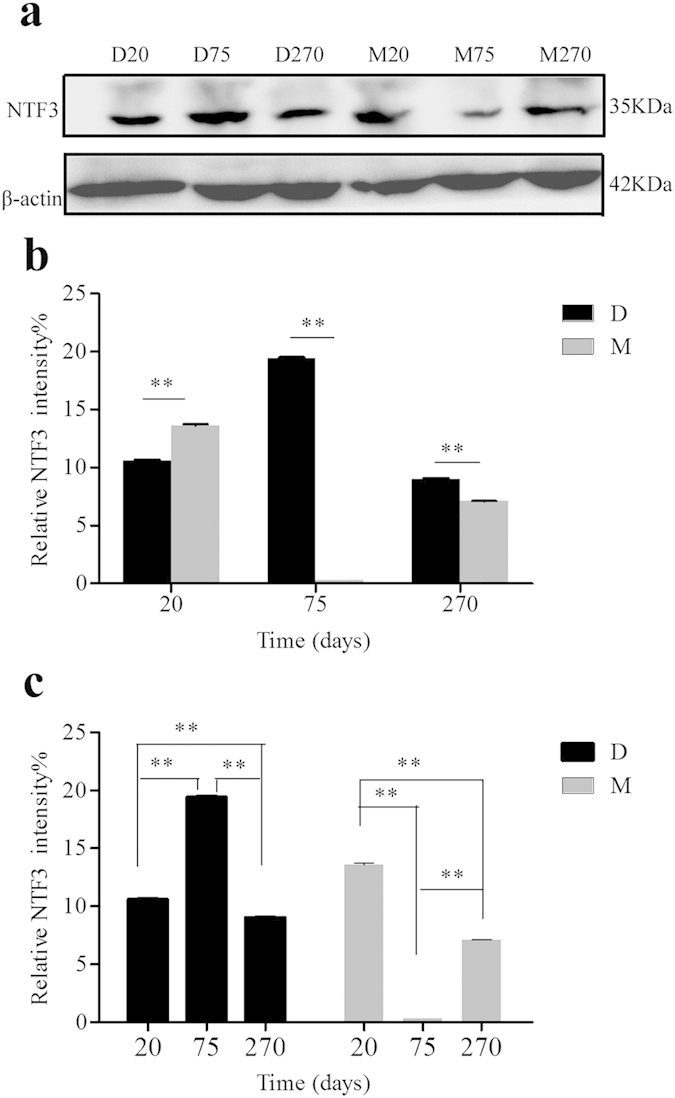
Expression of NTF3 in Duroc and Meishan boars testes at different ages. (**a**) Results of Western bloting, lanes from left to right represent the testes of 20-day-old, 75-day-old, 270-day-old Duroc boars and 20-day-old, 75-day-old, 270-day-old Meishan boars. (**b**,**c**) Relative expression level of NTF3 protein was detected with ImageJ, the experiments were repeated three times, the value expressed by each bar represents the mean ± SD. Student’s t-test was used for statistical significance (**p < 0.01).

NTF3 can induce cell migration from the early mesonephros into the developing testis, which is critical to the early morphological events of testis cord formation^22^. To verify the differential expression of NTF3 at the protein level (as shown in Fig. 5 and Supplementary Fig. S7 online), we isolated proteins from testes tissues. The expression of NTF3 in different samples at the RNA level was consistent with the expression at the protein level, with the exception that the expression at the mRNA level decreased from M20 to M270, whereas the protein expression level was high in M270. These results may be attributed to post-transcriptional regulation. The comparison between the breeds showed that the expression level of NTF3 in M20 was significantly higher than D20; in addition, the expression was significantly lower in M75 and M270 compared with D75 and D270 at the same stage. A within-breed comparison showed that NTF3 was significantly up-regulated at the protein level from 20 to 75 days and down-regulated from 75 to 270 days in the Duroc boars. Conversely, the lowest expression level was observed in M75, and the highest expression level was observed in M20.

**Figure 6:**
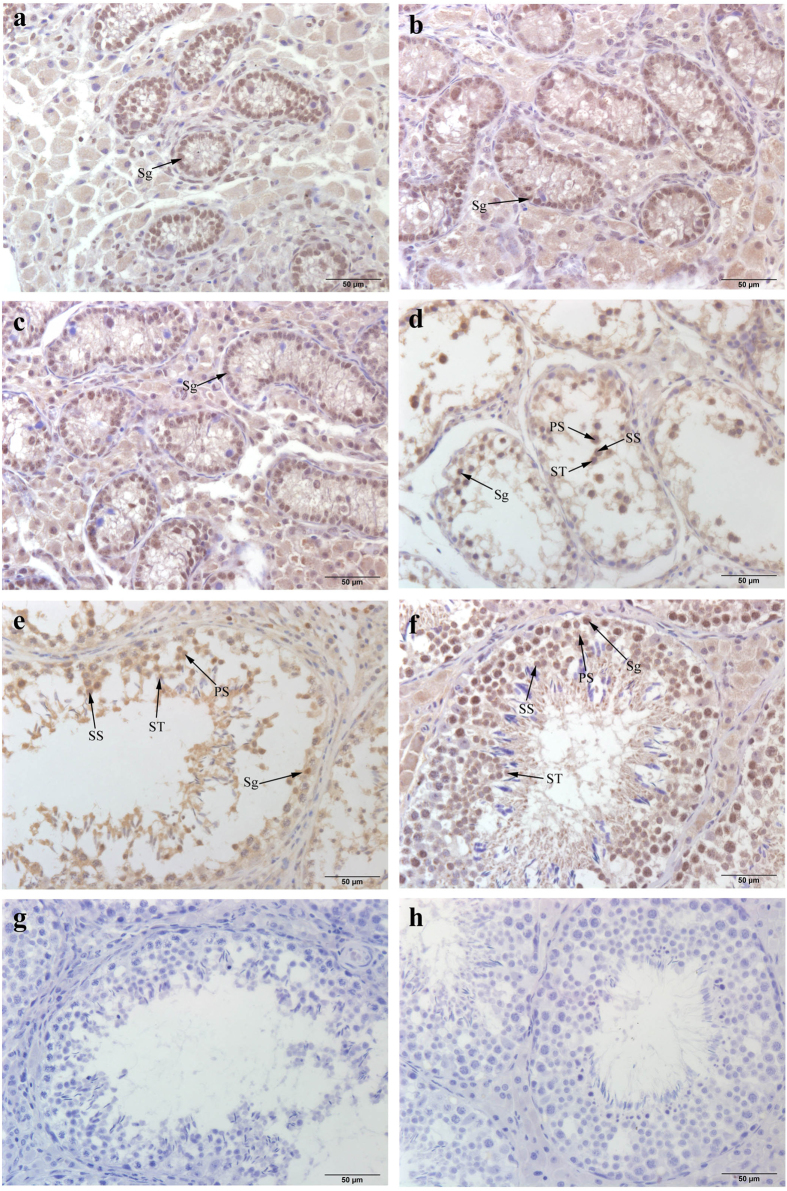
The location of NTF3 in Duroc and Meishan boars testes at different ages (immunohistochemical staining, 400×). Sg-spermatogonia, PS-primary spermatocyte, SS-secondary spermatocyte, ST-spermatid. (**a**) Testis of 20-day-old Duroc boar. (**b**) Testis of 20-day-old Meishan boar. (**c**) Testis of 75-day-old Duroc boar. (**d**) Testis of 75-day-old Meishan boar. (**e**) Testis of 270-day-old Duroc boar. (**f**) Testis of 270-day-old Meishan boar. (**g**) Control group of Duroc boar. (**f**) Control group of Meishan boar. In (**a**–**f**), anti-NTF3 was used as the primary antibody, and in (**g**–**h**), normal goat serum were used as a negative control. The brown signal in the images represents the location of NTF3, and the blue parts represent the nucleus, stained by Hematoxylin Staining Solution. Bars = 50 μm.

To identify the cellular localization of NTF3 in the boar testes, the localization of NTF3 was assessed using immunohistochemical staining (Fig. 6). NTF3 was localized on spermatogonia in the seminiferous tubule wall at 20 days based on staining using an NTF3-specific antibody. With the development of the testes, NTF3 expression decreased in spermatogonia and began to be expressed in spermatocytes and spermatids after sexual maturity was attained.

## Discussion

In this study, we conducted histological and RNA-seq analyses of the testes from Chinese Meishan and Duroc boars. Although some reproductive characteristics in boars have been conserved during thousands of years of selection, breed differences exist in body size, testis size, and number of sperm per ejaculate. The testis volume (p < 0.05) in Meishan boars was smaller than that in Duroc boars, and the total sperm per ejaculate in Duroc boars was higher (p < 0.05) than in Meishan boars^3^. Previous studies of Sertoli cell development in pigs indicated that the early postnatal period corresponded to the most rapid rate of Sertoli cell proliferation^2^,^8^. During the first month life a 6-fold increase in the number of Sertoli cells was found^23^ and approximately 15–30% of the Sertoli cells population were undergoing proliferation between 1 and 14 days of age^24^. In addition, the first oestrus occurred during the age range of 56–84 days in Chinese Meishan boars compared with 120–180 days for conventional boars^1^; however, both breeds attained sexual maturity by 270 days of age. Therefore, this study selected boars at 20, 75 and 270 days as our research materials. We conducted histological analyses and determined that the seminiferous tubule diameters of Meishan boars was less than Durocs at 20 days but it was greater than those in Duroc boars at 75 days due to the rapid development. The increasing number and diameters of seminiferous tubules determined the initiation of male puberty^8^. Puberty was initiated in M75, whereas no spermatocytes and spermatids were detected in D75 (Fig. 1). These results demonstrated that puberty occurred prior to 75 days in Meishan boars. In boars, sperm production is primarily determined by the number of Sertoli cells, which establishes testicular weight^10^. Histological analyses revealed that the number of Sertoli cells rapidly proliferated prior to 20 days and reacrched a stable state at 20 and 75 days in the Meishan boars but decreased from 75 to 270 days (Fig. 2b). Conversely, the Sertoli cells rapidly proliferated from 20 to 75 days and attained a stable state when the Durocs were 270 days old. Therefore, the rapidly proliferation of Sertoli cells occurred at an earlier stage and sustained a shorter time in the Meishan boars. A previous study showed that Sertoli cells were involved in the formation of the blood-testis barrier, which appeared at 40 days in the Meishan boars and 90–120 days in the Duroc boars^1^. This finding was consistent with our results, which indicated the stable phase (20–75 days) occurred at an earlier stage in the Meishan boars. Therefore, this study demonstrated that neither boar breed attained puberty at 20 days and that the number of Sertoli cells and spermatogonia significantly increased (p < 0.01) in the Meishan boars. The Meishan boars attained puberty and sperm appeared in their testes prior to 75 days; these results were not observed for the Duroc boars. An increased (p < 0.01) amount of spermatogonia and a larger (p < 0.01) diameter were observed for the Meishan boars at 75 days. The two species were in adulthood when they were 270 days old, and fewer (p < 0.01) spermatogonia were observed in the Meishan boars compared with the Duroc boars. Thus, the histological analysis demonstrated the early sexual maturity of Chinese Meishan boars.

The transcriptome data that were obtained in this study are useful for the elucidation of testis development in different stages between Duroc boars and Meishan boars. It has reported that more than 18 M reads from RNA-seq analyses are required for each sample to attain a saturated state for novel gene discovery and expressional analysis^16^,^19^. Our quality control assays for our data (including the randomness assessment, the base sequence quality and the distribution and coverage analyses in reference genes and genomic scaffolds) revealed that our RNA-seq data were well qualified (see Supplementary Table S2 online).

Bioinformatics analyses of DEGs revealed many DEGs between the breeds or between the different developmental stages. After strict filtering with our standard, 190 significant DEGs were obtained from six samples, as presented in Fig. 3b. We obtained two groups: M20, D20 and D75 clustered and M75, M270 and D270 clustered. This finding was in accordance with our histological analyses. The expression pattern for M75 was similar to the expression pattern for M270 and D270, which were in adulthood. These results were consistent with early sexual maturity of Meishan boars, which attained a mature status at 75 days. A large number of genes were down-regulated in M20, D20 and D75 but up-regulated in M75, M270 and D270. These genes may serve crucial roles in regulating testis development, and their expression may induce sexual maturity and spermatogenesis. Some of them are also detected by proteomics analysis, such as glutathione peroxidase 4 (GPx4), glutathione S-transferase mu 3 (GSTM3)^25^ and cytochrome b-c1 complex subunit 1 (UQCRC1)^26^, which related to male fertility.

The pathway analysis of significant DEGs, which based on the Kyoto Encyclopaedia of Genes and Genomes (KEGG) database, clustered the genes into seven pathways that regulated boar sexual function. Wnt signaling serves an essential role during testis development and can impact testis-related disorders^27^. Many functional genes related to spermatogenesis or reproduction in pigs involve the p53, mitogen-activated protein kinases (MAPK) and Wnt signaling pathways^28^. The mammalian target of rapamycin (mTOR) pathway was involved in regulating the proliferation of rat Sertoli cells^29^. The prostate is one of the male accessory sex organs; prostate cancer significantly influences the reproductive system and is among the most frequently diagnosed solid tumours among men^30^. Steroidal testosterone depletion retards testes growth, reduces the relative weight of the testes and accessory sex organs, and reduces sperm counts and motility^31^. We detected a large number of DEGs in the pathways between the two breeds at different ages, as shown in Table 1, which indicated many DEGs were involved in the MAPK pathway. We also annotated the GnRH-mediated and epigenetic regulation pathways and verified their involvement in the regulation of sexual maturity development^32^,^33^. The MAPK and GnRH signaling pathways can function together to regulate mammalian reproduction^34^. MAPK activity regulated by GnRH is necessary for normal fertility. A previous study reported that *in vivo*, conditional, and pituitary-specific disruption of ERK (extracellular signal-regulated kinase) signaling by GnRH caused to a gender-specific perturbation of fertility^35^. Epigenetic regulation is one of the major factors that regulate gene expression in response to environmental stimuli; this regulation involves chemical modification of DNA cytosine residues without altering the DNA sequence^19^. Aberrant DNA methylation patterns of spermatozoa in men affect fertility and sperm function^36^. Our RNA-seq data identified many epigenetic genes that were significantly differentially expressed between Duroc and Meishan boars at different ages. We speculated that the genes also served critical roles in inducing differences in male fertility between Duroc and Meishan boars and between different ages within the breed, as DNMT3A conditional mutant males were previously demonstrated to exhibit impaired spermatogenesis^37^.

Alternative splicing is one of the most important RNA modifications. This process causes protein diversification and contributes to the complexity of higher organisms^38^,^39^.This study detected four primary alternatively spliced events. We also detected a large proportion of genes that contain one or more alternatively spliced events from the six samples (see Supplementary Fig. S4 online). Alternative splicing is considered to be a key factor that increases cellular and functional complexity in eukaryotes^40^. SNPs were detected in coding sequence; most of the genomic variation was attributed to SNPs, which may also increase the functional complexity and offer the highest resolution for tracking disease genes and population history^41^. Approximately 60,000–140,000 SNPs from each sample were identified based on specific matching to the *Sus scrofa* genome, and the numbers of SNPs in the Meishan boars were greater than in the Duroc boars. The SNP data are useful for the identification of candidate genes and biomarkers for boar fertility^15^,^42^. But the SNPs in this study were only collected from coding DNA sequences because RNA-seq data only covers coding DNA regions. A larger number of SNPs may exist in the non-coding sequences compared with the coding sequences. It deserves further investigation by whole-genome sequencing.

QTLs that affect the age of porcines at puberty are located in 19 chromosomes (based on the pig QTLdb and shown in Table S4), and a larger number of DEGs located in the QTL are shown in Table 2a. The selecting of candidate genes to explore the regulatory mechanism of puberty will be the subject of a future study. After searching for new differentially expressed transcripts and genes in QTLs related to reproduction traits (as shown in Table 2b), we determined that the number of significant DEGs and novel transcripts between breeds at 75 days was substantially larger than the same number at 20 and 270 days. More DEGs were detected between 20 and 75 days in Meishan boars, which validated the differences in the reproductive traits between Meishan and Duroc boars.

To validate the reliability of the data, we selected important DEGs related to reproduction and performed an RT-PCR experiment. The RT-PCR results matched the RNA-seq data (see Supplementary Fig. S6 online). NKX3-1 in the mature testis can cause spermatogenic cell division and differentiation and prompt spermatogenesis if it is suppressed^43^. A previous study demonstrated that increased PTEN may decrease spermatogenesis, fertility, and sperm maturation, and fertilization in the Akt1−/− and Akt2−/− male mice^44^. The receptor GnRHR2 is involved in the GNRH signaling pathway. Several intron retention events and a few SNPs were detected in GnRHR2, which may link its ligand GnRH to the regulation of LHβ release and reproduction^45^. We identified genes related to spermatogenesis and the regulation of mammalian reproduction. Therefore, it was worthwhile to validate the expression of the genes. CTNNB1, NTF3 and NR0B1 are considered to be testis-promoting, differentaition and maintenance genes, and CTNNB1 can cause aberrant testis development if mutated^46^. This study also showed that CTNNB1 had three alternative splicing events, including IR, A3SS and A5SS. We also found SNP sites from CTNNB1 in D20, D75 and D270, with the exception of the Meishan boars. Therefore, whether CTNNB1 is a critical gene for the sexual differences between Meishan boars and Duroc boars warrants further study. NTF3 was involved in the progression of male sex differentiation and was critical to the induction of embryonic testis cord formation^46^,^47^. NTF3 was involved in the MAPK signaling pathway, which was involved in the regulation of mammalian reproduction^34^. Our study showed that NTF3 was significantly differently expressed between Duroc and Meishan boars at different ages and that its expression decreased at adulthood, which suggests that NTF3 also served an important role in the early development of testes. An immunohistochemical assay indicated NTF3 was localized on E14 and E16 in embryonic rat testis Sertoli cells and was expressed in germ cells and Leydig cells at E16^22^. Our study demonstrated NTF3 was primarily localized in spermatogonia and was detected at low levels in the spermatocyte and sperm; however, no expression was detected in the Sertoli cell after birth. Therefore, we deduced that NTF3 also served a significant role in postnatal spermatogenesis. NTF3 protein expression showed an expression pattern that was similar to the RNA expression. However, NTF3 was significantly up-regulated at the protein level in D270 compared with the D75. One explanation may be that post-transcriptional regulation caused the different expression patterns between the RNA and protein levels.

Histological analyses illustrated that Meishan boars underwent puberty and sexual function development at an earlier stage than Duroc boars. RNA-seq enabled us to obtain potential signature genes and discover pathways that were significantly involved in the regulation of male sexual function. A large number of alternative splicing events and SNPs that detected in the testes require further analysis. The analyses of DEGs and QTLs related to reproduction aided in our understanding of the molecular mechanisms that control the sexual development of Duroc and Meishan boars. This study will provide a valuable transcriptomics resource for Duroc and Meishan boars and enable researchers to identify functional genes, molecular markers and pathways that influence sexual function development and spermatogenesis.

## Methods

### Animals and Samples Collection

All animals were raised under the same conditions. We obtained 20-, 75- and 270-day-old clinically healthy Duroc and Chinese Meishan boars from the Fine Farm of Hua Zhong Agricultural University. Tissue samples from testes were frozen in liquid nitrogen immediately after collection and stored at −80 °C prior to RNA extraction. A proportion of the samples from the testes were fixed with 4% formaldehyde (40% Formaldehyde solution: distilled water in a 1:9 ratio) solution for histological comparison. The methods were performed in accordance with the approved guidelines from Huazhong Agricultural University, and scientific, ethical and legal principles of the Hubei Regulations for the Administration of Affairs Concerning Experimental Animals. All experimental protocols were approved by the Ethics Committee of Huazhong Agricultural University.

### Light Microscope Observation and Statistical analysis

Materials were fixed in 4% formaldehyde solution at room temperature. The samples were washed with running water, dehydrated using an ethyl alcohol series, cleared in xylene and embedded in paraffin wax. The specimens were sectioned to a thickness of 4 μm using a Leica RM2235 microtome (Leica Instrument Company, Germany). Cross-sections were stained with HE, examined and photographed using an Olympus BX51 (Olympus Optical Co. Japan) biomicroscope. Seminiferous tubules were photographed using an Olympus BX51 and digital imaging system (DP72).The diameters of seminiferous tubules were measured using the Image Pro-Plus software at 400× magnification in 30 randomly selected cross-sections. The number of spermatogonia and Sertoli cells were also measured using Image Pro-Plus from 30 seminiferous tubule cross-sections at 200× magnification. Significance analyses that pertain to the diameters of the seminiferous tubules were conducted using a T-test.

### Library Preparation and Quantifying

Total RNA was extracted from the testes of 20-, 75- and 270-day-old Duroc and Meishan boars (designated D20, D75, D270, M20, M75 and M270) using TRIzol reagent (Invitrogen) according to the manufacturer’s instructions. The samples were sent to Shenzhen Genomics Institute (BGI) with drikold. The RNA integrity and concentration were evaluated with a NanoDrop 6000 Labchip kit (Nanodrop, Wilmington, DE, USA) and an Agilent 2100 Bioanalyzer (Agilent Technologies, Santa Clara, CA, USA). An RNAeasy MiniElute Cleanup kit (Qiagen, Valencia, CA, USA) was used for RNA purification to obtain six RNA pools. Construction of the sequencing libraries was performed using the Illumina mRNA-seq Sample Preparation kit (Illumina, San Diego, CA, USA) according to the manufacturer’s instructions. Sequencing was performed using the Illumina Genome Analyzer (Illumina, San Diego, CA, USA). Approximately 70–83 M clean reads per sample were generated for the genome-wide transcriptomic analyses.

### Mapping and Assembling

The trimmed reads were assembled and mapped to the reference genome (Sscrofa10.2) and reference gene (ftp://ftp.ncbi.nlm.nih.gov/gene/) collections by performing alignments using the SOAPaligner/SOAP2 software^48^ (-m 0 -x 1000 -s 40 -l 32 -v 5 -r 2 -p 6). Alignment with reference sequences was performed to discover new transcripts with length ≥180 bp and sequence depths ≥2 with distance within 200 bp of the annotated gene^49^. Alternative splicing and SNP analyses were performed using the SOAPsplice^50^ (-g 1 -m 3 -p 6) and SOAPsnp^51^ (-u -t -Q i -L 90) software, respectively.

### Differential gene expression analysis

Values of RPKM were generated and used to identify the total number of genes expressed in each porcine sample and the DEGs among each comparison^52^. The DEGs between two samples were analysed based on an algorithm as previously described^52^. The P-value corresponds to a differential gene expression test in which False Discovery Rate (FDR) was used to determine the threshold of the P-value in multiple tests. The Cluster 3.0^53^ was used for the clustering analysis with parameters “-g 7 -e 7 -m a”. The R heatmap package^54^ was applied to the analysis of Pearson and Spearman clustering. The functional classification of genes was performed using KEGG^55^ pathway analysis.

### Search for differentially expressed new transcripts and DEGs in QTL regions related to reproduction

We filtered the DEGs with the standards (FDR < 0.001, |log2Ratio|>1) and RPKM > 1. According to the location information of the reference genome (Sscrofa10.2) and pig QTL dababase (http:// www.Animalgenome.org/QTLdb/), differentially expressed new transcripts and known genes were identified in the QTL regions or at the boundary.

### RT-qPCR validation of the DEGs

Eight genes were chosen for confirmation of the DEG data by qRT-PCR. Aliquots of total RNA extracted for sequencing, as previously described, were used for the qRT-PCR experiments according to the manufacturer’s instructions (Roche, Shanghai, China). All qRT-PCR experiments were run in a 10 ul reaction volume with the Roche LightCyler 480 system (Roche). The primers for the RT-PCR assays are listed in Table S1. The β- actin gene was used as a constitutive expression control in these experiments. The relative gene expression data were analysed using the 2^−ΔΔCt^ method^56^. All reactions were performed in triplicate. The relative expression values were then compared with the RNA-seq data.

### Western blotting

Tissues were added to cell lysis buffer (Radio immunoprecipitation assay (RIPA):phenylmethanesulfonyl fluoride (PMSF) in a 100:1 ratio) after they were ground in liquid nitrogen. Then, the samples were heated to 99 °C for 10 min in 5× SDS buffer, separated by SDS-PAGE, and transferred to PVDF membranes (Millipore). Then the membranes were blocked with 5% non-fat dried milk and incubated overnight with anti-NTF3 (Santa Cruz). β-actin (Santa Cruz) was used as a loading control. After washing, the membranes were incubated with a horseradish peroxidese-conjugated anti-rabbit antibody (Santa Cruz; 1:4000) for 1.5 h at room temperature and visualized using the ECL Western Blotting Detection System (Tiangen).

### Immunohistochemistry

Testis sections from 20-, 75- and 270-day-old Duroc and Meishan boars were deparaffinized and rehydrated with xylene and a graded ethanol series, respectively. When antigen retrieval was achieved, the sections were blocked 30 min in 3% hydrogen peroxide, boiled in 0.01 M sodium citrate buffer in a microwave on high for 5 min, boiled at a low setting for 20 min to expose the antigens, and washed with PBS. Then, the samples were blocked with 10% fatal bovine serum (Gibco) for 2 h prior to incubation with 0.5 μg/ml of primary anti-NTF3 antibody for 12 h (Santa Cruz Biotechnology, Santa Cruz, CA, USA, sc-547). Negative control experiments were performed using 10% fatal bovine serum. The sections were washed with PBS and incubated with a secondary antibody (Santa Cruz 1:300) for 30 min; then, the samples were washed with PBS again. Diaminobenzidine staining was performed with the NTF3 localization for 3–10 min, and hematoxylin staining was performed for 1–5 min. Differentiation was assessed with 0.5% hydrochloric acerbic for 15 s. Then the sections were dehydrated using an ethyl alcohol series, cleared in xylene, embedded in paraffin wax, and photographed using a microscope (Olympus BX51).

### Statistical analyses

All experiments were repeated at least three times. Data were given as mean ± SD. Student’s t-test was used for statistical comparisons. A p value < 0.05 was considered to be statistically significant.

## Additional Information

**How to cite this article**: Ding, H. *et al.* Histological and transcriptome analyses of testes from Duroc and Meishan boars. *Sci. Rep.* **6**, 20758; doi: 10.1038/srep20758 (2016).

## Supplementary Material

Supplementary Information

## Figures and Tables

**Table 1:** The number of all genes and DEGs annotated in the pathways.

Pathway	All genes with pathway annotation	DEGs with pathway annotation		
		D20-vs-M20	D75-vs-M75	D270-vs-M270
GnRH signaling pathway	213	49	72	33
MAPK signaling pathway	514	99	187	92
Wnt signaling pathway	300	51	112	46
mTOR signaling pathway	99	14	41	11
p53 signaling pathway	161	37	68	34
Prostate cancer	170	28	69	33
Steroid biosynthesis	29	12	9	7

The seven pathways are related to boar sexual function.

**Table 2:** The number of differentially expressed new transcripts and genes.

Traits	QTL	Differentially expressed genes	Differentially novel transcripts
(**a**)			
Age at puberty	AGEP	979	6807
Epididymis weight	EPIDW	198	1057
Seminiferous tubule diameter	SEMTD	2	92
Ejaculation duration	EJDUR	1	34
Semen PH	SEPH	3	19
Sperm concentration	SPCON	1	13
Sperm motility	SPMOT	4	0
Sperm per ejaculate	SPEEJ	7	0
Testicular weight	TWSTIWT	7	0
Semen volume	SEMVOL	0	43
Ejaculation times	EJTIME	0	23
Sperm abnormality rate	SPABR	0	5
(**b**)			
**Samples**	**Differentially expressed genes**	**Differentially novel transcripts**	
D20-VS-M20	298	1285	
D75-VS-M75	642	3867	
D270-VS-M270	284	1615	
D20-VS-D75	223	711	
D20-VS-D270	708	5393	
D75-VS-D270	656	5148	
M20-VS-M75	659	3285	
M20-VS-M270	843	5339	
M75-VS-M270	664	3736	

(**a**) The number of differentially expressed new transcripts and genes in QTL regions related to reproduction traits. (**b**) The number of differentially expressed new transcripts and genes in QTL regions from each comparison.
