# Supplementary material for: Histological and transcriptome analyses of testes from Duroc and Meishan boars
Source: Sci Rep. 2016 Feb 11;6:20758. doi: 10.1038/srep20758 (PMC4749976; doi:10.1038/srep20758)
Supplement: Supplementary Information [file srep20758-s1.pdf]

# Histological and transcriptome analyses of testes from Duroc and Meishan boars

Haisheng Ding[1,+], Yan Luo[1,+], Min Liu[1,*], Jingshu Huang[2], Dequan Xu[1,*]

[1]Key Laboratory of Swine Genetics and Breeding of Ministry of Agriculture, and Key Lab of Agricultural Animal Genetics, Breeding and Reproduction of Ministry of Education, Huazhong Agricultural University, Wuhan 430070, China

[2]Animal Husbandry and Veterinary Bureau of Hubei Province, Wuhan 430064, China.

[*]Corresponding author:liumin23@mail.hzau.edu.cn (Min Liu); dequanxu@126.com (Dequan Xu)

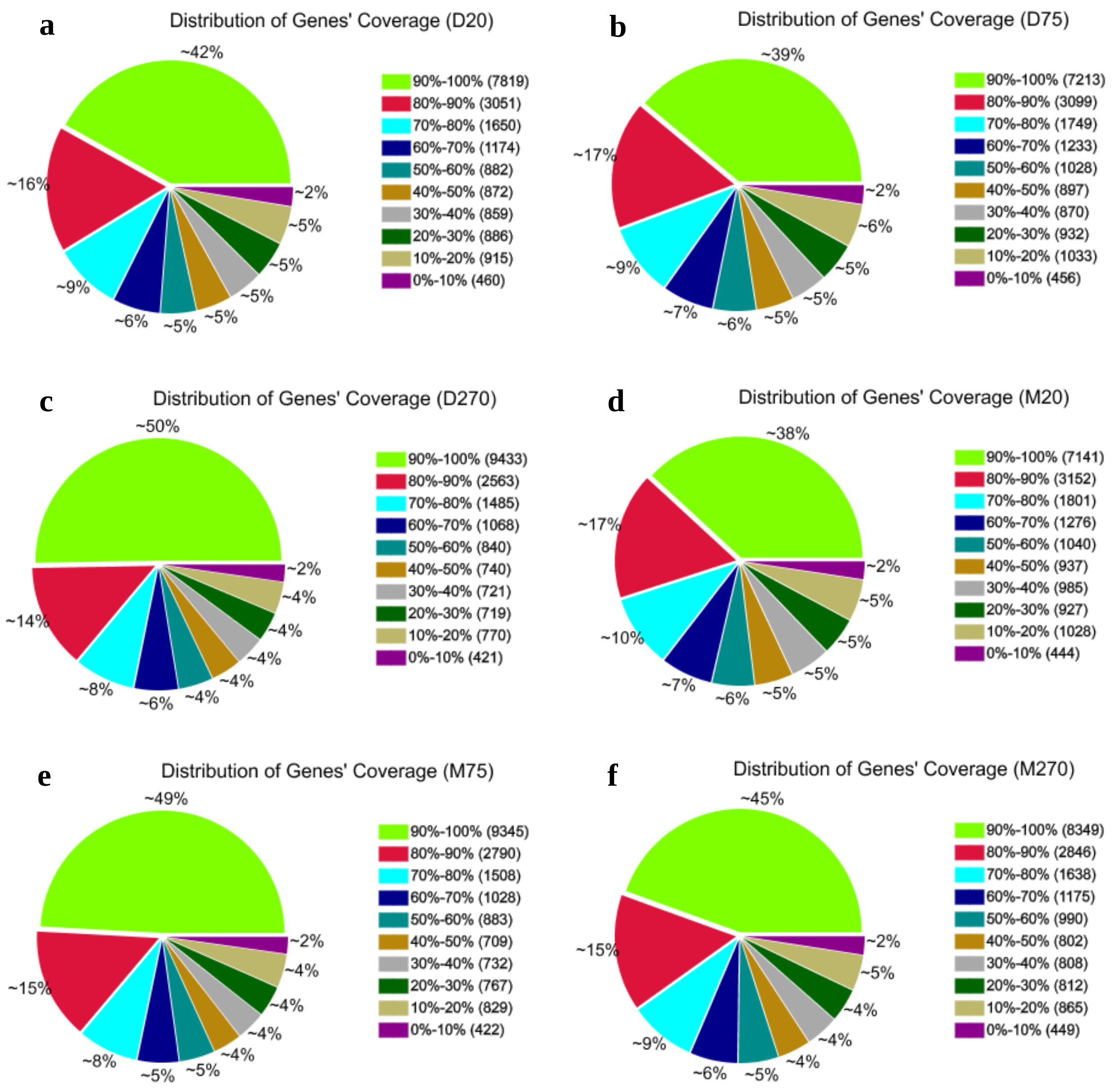


**Supplementary Figure S1. Distribution of read coverage in transcripts detected.** (a) D20. (b) D75. (c) D270. (d) M20. (e) M75. (f) M270.

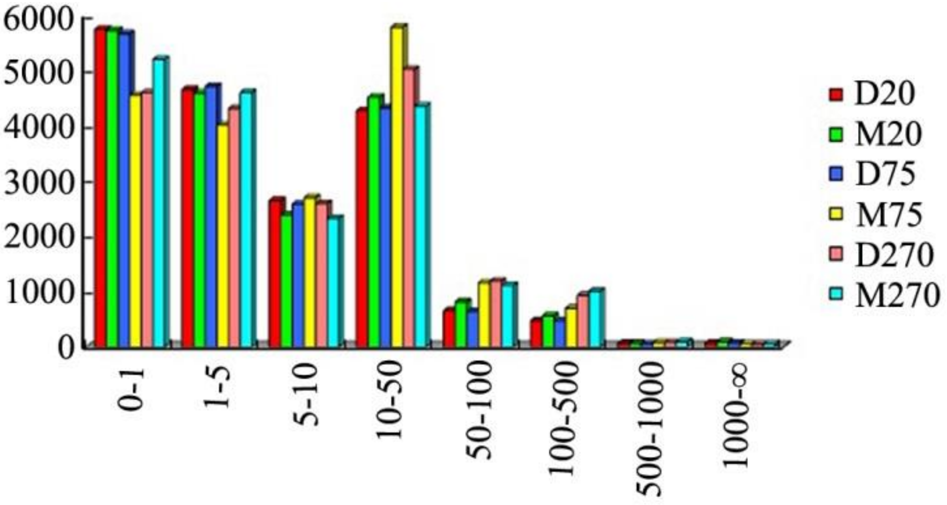


**Supplementary Figure S2. Abundant distribution of genes.** 0-10, low expression genes. 10-500, moderate expression genes. >500, high expression genes.

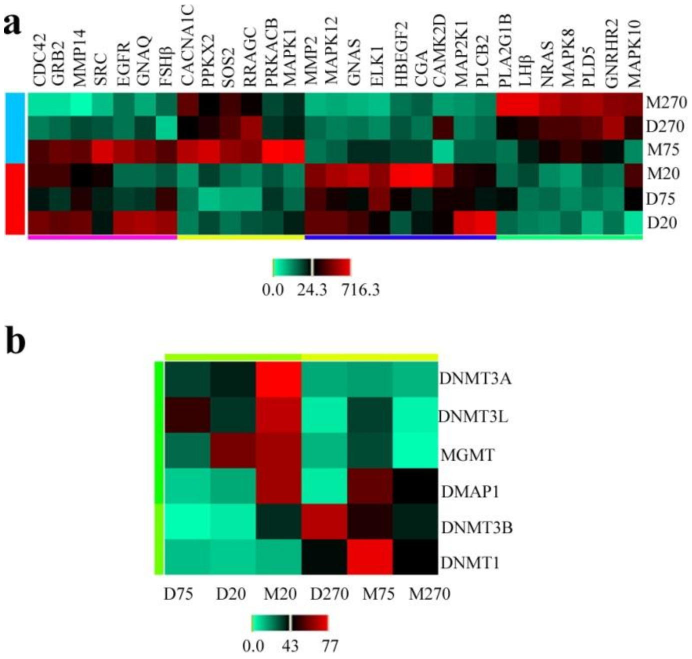


**Supplementary Figure S3. Heatmap results.** (a) Heatmap of the subset DEGs in the GnRH pathway from D20-vs-M20, which is critical to reproduction regulation. (b) Heatmap of DEGs encoding key enzymes in epigenetic regulation during spermatogenesis.

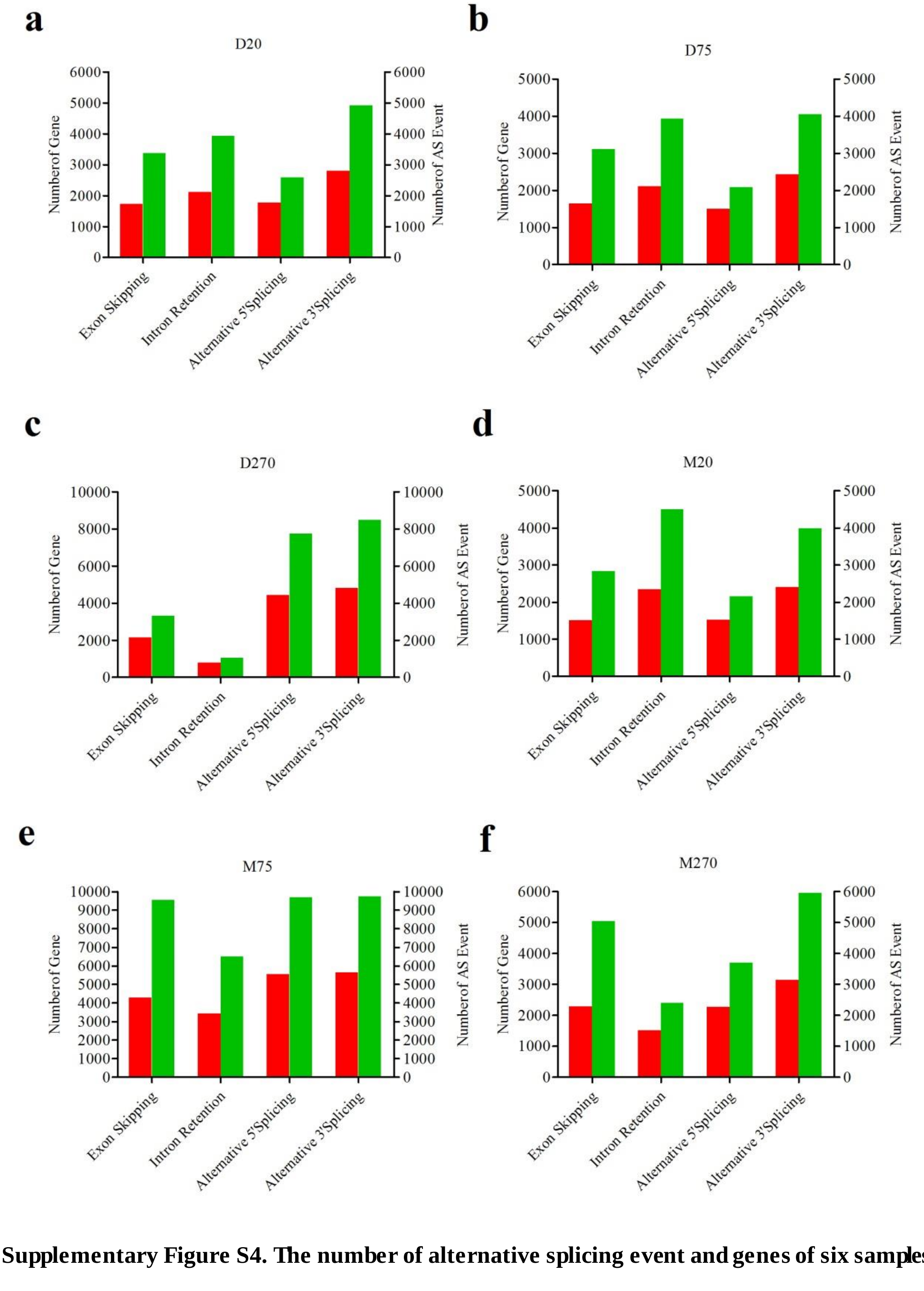


**Supplementary Figure S4. The number of alternative splicing event and genes of six samples.** (a) D20. (b) D75. (c) D270. (d) M20. (e) M75. (f) M270.The red bars indicated the number of genes and the green bars indicated the number of alternative splicing events. AS, alternative splicing.

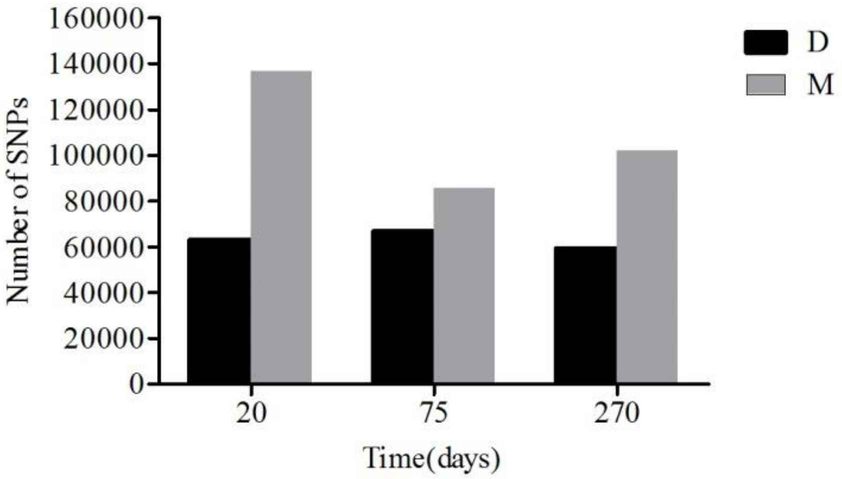


**Supplementary Figure S5. The number of SNPs in six samples.** The black bars show the number of SNPs in Duroc boars, and the gray bars show the number of SNPs in Meishan boars. D indicates Duroc boars; M indicates Meishan boars.

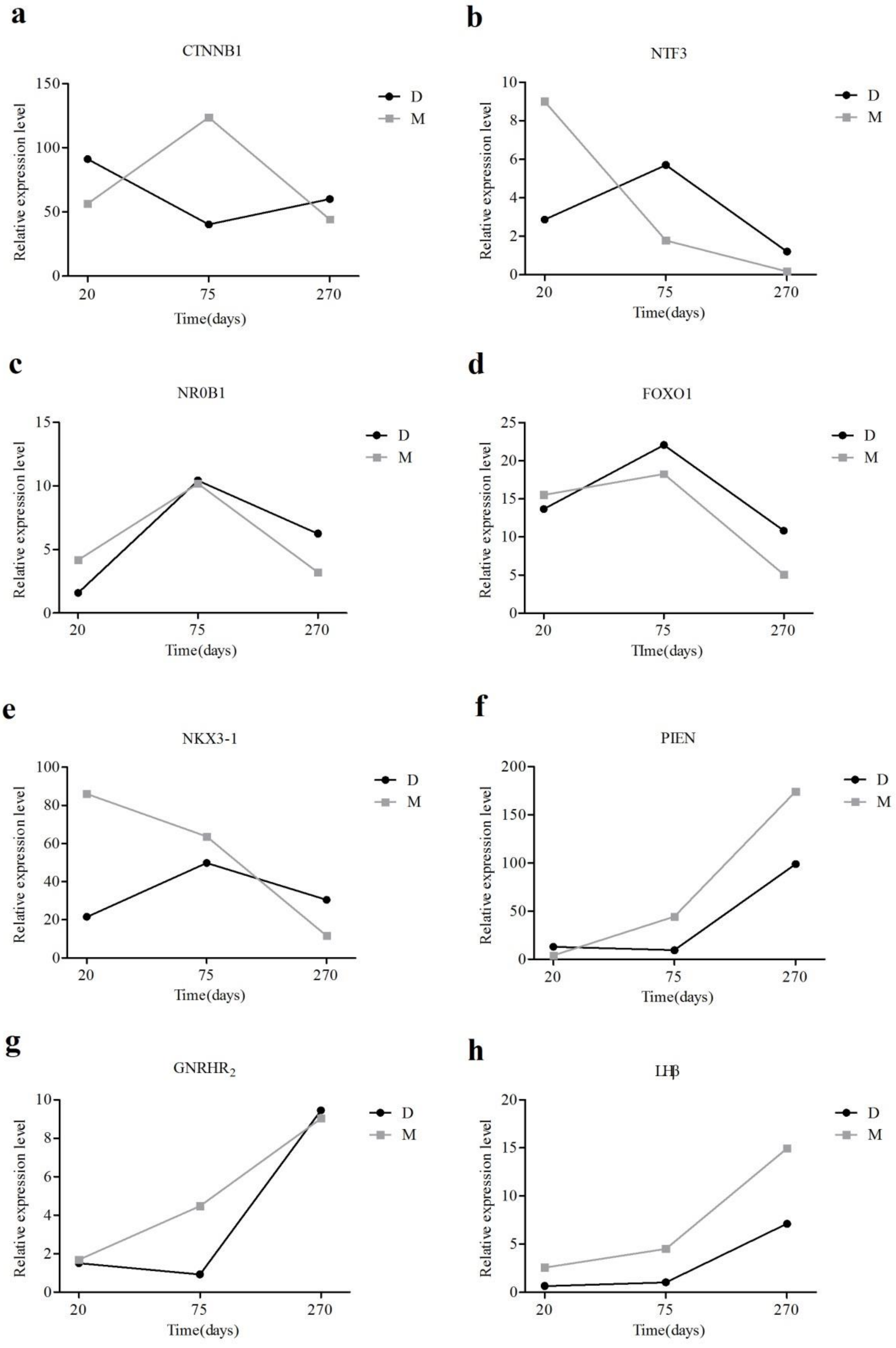


**Supplementary Figure S6. The results of RNA-seq between the two breeds.** (a) CTNNB1. (b) NTF3. (c) NR0B1. (d) FOXO1. (e) NKX3-1. (f) PTEN. (g) GNRHR$_2$. (h) LHβ. D indicates Duroc boars; M indicates Meishan boars.

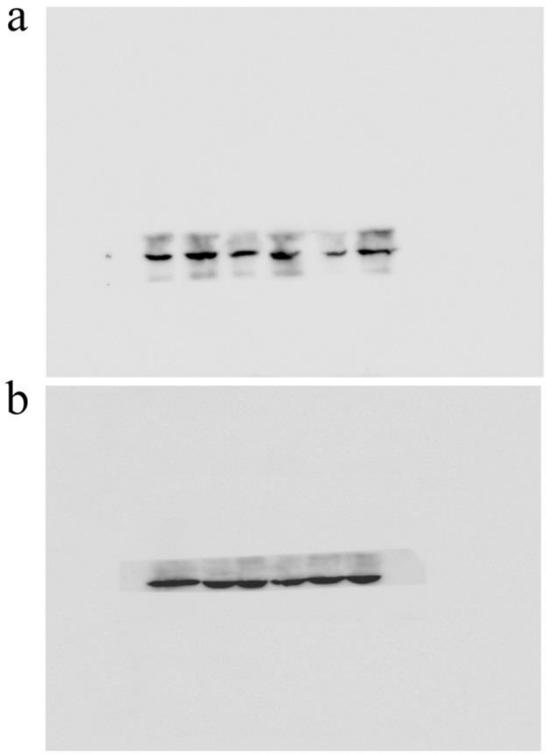


**Supplementary Figure S7. Full image of Western Blotting.** (a) NTF3 was probed with anti-NTF3 antibody. (b) β-actin was probed with anti-β-actin antibody. Lanes from left to right represent the testes tissues of 20-day-old, 75-day-old, 270-day-old Duroc boars and 20-day-old, 75-day-old and 270-day-old Meishan boars.

**Supplementary Table S1. Primers for real-time qPCR.** [1]F, forward primer. [2]R, reverse primer.

| Primer | Sequence of primers (5'-3') | Tm(°C) | Size (bp) |
|---|---|---|---|
| CTNNB1-F[1] | TAAACTCCTACATCCACCATC | 57 | 141 |
| CTNNB1-R[2] | CGAACCAGCAACTGAACT | | |
| NKX3-1-F | AGGGAGGAAGAGGCAGCG | 65 | 128 |
| NKX3-1-R | GTCCAGCAGATAAGTCTCAAGGTG | | |
| PTEN-F | TATTCCCAGTCAGAGGCG | 61 | 215 |
| PTEN-R | CGAACTTGTCTTCCCGTC | | |
| FOXO1-F | CAGAGGGAGGCAAGAGTG | 59 | 211 |
| FOXO1-R | GTCCAGTTATCAAAGTCATCG | | |
| NR0B1-F | CTTCCTTGCCAAGTGCTGGA | 63 | 179 |
| NR0B1-R | GGTACCCCCTGTGTGTCATC | | |
| NTF3-F | TATCACCCTGCCCAAAGC | 62 | 196 |
| NTF3-R | TCCCACATAATCCTCCACG | | |
| GnRHR2-F | CAGTTTCCTTCTTGCCTTGCC | 61 | 106 |
| GnRHR2-R | TTGAGCCTTGAAGCTGCCTT | | |
| LHβ-F | TAGGGACTGCTGTTGTGGCT | 62 | 146 |
| LHβ-R | ATGCTGGTGGTGAAGGTGAT | | |
| β-actin-F | CCAGGTCATCACCATCGG | 60 | 158 |
| β-actin-R | CCGTGTTGGCGTAGAGGT | | |

**Supplementary Table S2. Quality evaluation of transcriptome library.** (a) RNA quality detection for samples. RNA samples (n=3) of each library were mixed together when measured. (b) Statistics of transcriptome library in each sample. (c) The number of reads mapping to reference genome and reference gene from each sample.

| Sample | Concentration （(ng/ μL） | Quality (μg) | RIN | 28S/ 18S | OD260/280 | Test results |
|---|---|---|---|---|---|---|
| D20 | 1593.0 | 55.76 | 9.0 | 1.6 | 1.98 | A type |
| D75 | 2210.0 | 55.25 | 8.7 | 1.6 | 1.96 | A type |
| D270 | 1110.0 | 61.05 | 8.0 | 1.8 | 2.00 | A type |
| M20 | 2610.0 | 78.30 | 8.0 | 1.6 | 2.00 | A type |
| M75 | 3069.0 | 138.11 | 8.7 | 2.0 | 1.99 | A type |
| M270 | 444.0 | 22.20 | 7.5 | 1.6 | 2.00 | A type |

**(a)**

| Sample | Raw reads | Clean reads |
|---|---|---|
| D20 | 88239426 | 82204520 (93.16%) |
| D75 | 82447852 | 78732460 (95.49%) |
| D270 | 84540534 | 79277080 (93.77%) |
| M20 | 88198764 | 82280956 (93.29%) |
| M75 | 75184178 | 70130324 (93.28%) |
| M270 | 89026326 | 78512750 (88.19%) |

**(b)**

| Sample | Total reads | Map to Genome | | Map to Gene | |
|---|---|---|---|---|---|
| | | Total mapped | Unique mapped | Total mapped | Unique mapped |
| D20 | 82204520 | 49006496（59.62%） | 45637349（55.52%） | 48794808（59.36%） | 46030981（56.00%） |
| D75 | 78732460 | 45998100（58.42%） | 42530620（54.02%） | 41090470（52.19%） | 38812571（49.30%） |
| D270 | 79277080 | 42098395（53.10%） | 38237952（48.23%） | 42366329（53.44%） | 39639577（50.00%） |
| M20 | 82280956 | 42409303（51.54%） | 38399404（46.67%） | 37433323（45.49%） | 34597892（42.05%） |
| M75 | 70130324 | 35668778（50.86%） | 32257507（46.00%） | 39153887（55.83%） | 36486454（52.03%） |
| M270 | 78512750 | 39422130（50.21%） | 35531534（45.26%） | 41166231（52.43%） | 38340413（48.83%） |

**(c)**

**Supplementary Table S3. The reads mapping to reference genome and reference gene from six samples.** (a) D20. (b) D75. (c) D270. (d) M20. (e) M75. (f) M270.

| D20 | Map to Genome | | Map to Gene | |
|---|---|---|---|---|
| | reads number | percentage | reads number | percentage |
| Total Reads | 82204520 | 100.00% | 82204520 | 100.00% |
| Total BasePairs | 7398406800 | 100.00% | 7398406800 | 100.00% |
| Total Mapped Reads | 49006496 | 59.62% | 48794808 | 59.36% |
| perfect match | 34381852 | 41.82% | 37004468 | 45.02% |
| <=5bp mismatch | 14624644 | 17.79% | 11790340 | 14.34% |
| unique match | 45637349 | 55.52% | 46030981 | 56.00% |
| multi-position match | 3369147 | 4.10% | 2763827 | 3.36% |
| Total Unmapped Reads | 33198024 | 40.38% | 33409712 | 40.64% |

**(a)**

| D75 | Map to Genome | | Map to Gene | |
|---|---|---|---|---|
| | reads number | percentage | reads number | percentage |
| Total Reads | 78732460 | 100.00% | 78732460 | 100.00% |
| Total BasePairs | 7085921400 | 100.00% | 7085921400 | 100.00% |
| Total Mapped Reads | 45998100 | 58.42% | 41090470 | 52.19% |
| perfect match | 34340094 | 43.62% | 33746382 | 42.86% |
| <=5bp mismatch | 11658006 | 14.81% | 7344088 | 9.33% |
| unique match | 42530620 | 54.02% | 38812571 | 49.30% |
| multi-position match | 3467480 | 4.40% | 2277899 | 2.89% |
| Total Unmapped Reads | 32734360 | 41.58% | 37641990 | 47.81% |

**(b)**

| D270 | Map to Genome | | Map to Gene | |
|---|---|---|---|---|
| | reads number | percentage | reads number | percentage |
| Total Reads | 79277080 | 100.00% | 79277080 | 100.00% |
| Total BasePairs | 7134937200 | 100.00% | 7134937200 | 100.00% |
| Total Mapped Reads | 42098395 | 53.10% | 42366329 | 53.44% |
| perfect match | 30844157 | 38.91% | 34042799 | 42.94% |
| <=5bp mismatch | 11254238 | 14.20% | 8323530 | 10.50% |
| unique match | 38237952 | 48.23% | 39639577 | 50.00% |
| multi-position match | 3860443 | 4.87% | 2726752 | 3.44% |
| Total Unmapped Reads | 37178685 | 46.90% | 36910751 | 46.56% |

**(c)**

| M20 | Map to Genome | | Map to Gene | |
|---|---|---|---|---|
| | reads number | percentage | reads number | percentage |
| Total Reads | 82280956 | 100.00% | 82280956 | 100.00% |
| Total BasePairs | 7405286040 | 100.00% | 7405286040 | 100.00% |
| Total Mapped Reads | 42409303 | 51.54% | 37433323 | 45.49% |
| perfect match | 23973246 | 29.14% | 23137340 | 28.12% |
| <=5bp mismatch | 18436057 | 22.41% | 14295983 | 17.37% |
| unique match | 38399404 | 46.67% | 34597892 | 42.05% |
| multi-position match | 4009899 | 4.87% | 2835431 | 3.45% |
| Total Unmapped Reads | 39871653 | 48.46% | 44847633 | 54.51% |

**(d)**

| M75 | Map to Genome | | Map to Gene | |
|---|---|---|---|---|
| | reads number | percentage | reads number | percentage |
| Total Reads | 70130324 | 100.00% | 70130324 | 100.00% |
| Total BasePairs | 6311729160 | 100.00% | 6311729160 | 100.00% |
| Total Mapped Reads | 35668778 | 50.86% | 39153887 | 55.83% |
| perfect match | 24172082 | 34.47% | 28600977 | 40.78% |
| <=5bp mismatch | 11496696 | 16.39% | 10552910 | 15.05% |
| unique match | 32257507 | 46.00% | 36486454 | 52.03% |
| multi-position match | 3411271 | 4.86% | 2667433 | 3.80% |
| Total Unmapped Reads | 34461546 | 49.14% | 30976437 | 44.17% |

**(e)**

| M270 | Map to Genome | | Map to Gene | |
|---|---|---|---|---|
| | reads number | percentage | reads number | percentage |
| Total Reads | 78512750 | 100.00% | 78512750 | 100.00% |
| Total BasePairs | 7066147500 | 100.00% | 7066147500 | 100.00% |
| Total Mapped Reads | 39422130 | 50.21% | 41166231 | 52.43% |
| perfect match | 25595635 | 32.60% | 29722518 | 37.86% |
| <=5bp mismatch | 13826495 | 17.61% | 11443713 | 14.58% |
| unique match | 35531534 | 45.26% | 38340413 | 48.83% |
| multi-position match | 3890596 | 4.96% | 2825818 | 3.60% |
| Total Unmapped Reads | 39090620 | 49.79% | 37346519 | 47.57% |

**(f)**

**Supplementary Table S4 . The QTLs related to reproduction traits and their chromosome positions**

| Traits | QTL | chromosome |
|---|---|---|
| Age at puberty | AGEP | chr1, chr2, chr3, chr4, chr5, chr6, chr7, chr8, chr9, chr10, chr11, chr12, chr13, chr14, chr15, chr16, chr17, chr18 |
| Epididymis weight | EPIDW | chr2, chr3, chr7, chrX |
| Seminiferous tubule diameter | SEMTD | chr5, chr13, chr16, chrX |
| Ejaculation duration | EJDUR | Chr6, chr17 |
| Semen PH | SEPH | chr2, chr6, chr9, chr12 |
| Sperm concentration | SPCON | chr1, chr5, chr17 |
| Sperm motility | SPMOT | chr1, chr5 |
| Sperm per ejaculate | SPEEJ | chr1, chr2 |
| Testicular weight | TWSTIWT | chr1, chr3, chr5, chr7, chrX |
| Semen volume | SEMVOL | chr1, chr3, chr15, chr18 |
| Ejaculation times | EJTIME | chr6, chr16, chr17 |
| Sperm abnormality rate | SPABR | chr4, chr5, chr9 |
